# A Novel Tigecycline Adjuvant ML-7 Reverses the Susceptibility of Tigecycline-Resistant *Klebsiella pneumoniae*

**DOI:** 10.3389/fcimb.2021.809542

**Published:** 2022-01-05

**Authors:** Lilan Sun, Lang Sun, Xue Li, Xinxin Hu, Xiukun Wang, Tongying Nie, Youwen Zhang, Xuefu You

**Affiliations:** Beijing Key Laboratory of Antimicrobial Agents, Institute of Medicinal Biotechnology, Chinese Academy of Medical Sciences & Peking Union Medical College, Beijing, China

**Keywords:** *Klebsiella pneumoniae*, resistance genes, tigecycline, ML-7, combination therapy

## Abstract

The increasing incidence of tigecycline resistance undoubtedly constitutes a serious threat to global public health. The combination therapies had become the indispensable strategy against this threat. Herein, 11 clinical tigecycline-resistant *Klebsiella pneumoniae* which mainly has mutations in *ramR*, *acrR*, or *macB* were collected for tigecycline adjuvant screening. Interestingly, ML-7 hydrochloride (ML-7) dramatically potentiated tigecycline activity. We further picked up five analogs of ML-7 and evaluated their synergistic activities with tigecycline by using checkerboard assay. The results revealed that ML-7 showed certain synergy with tigecycline, while other analogs exerted attenuated synergistic effects among tigecycline-resistant isolates. Thus, ML-7 was selected for further investigation. The results from growth curves showed that ML-7 combined with tigecycline could completely inhibit the growth of bacteria, and the time-kill analysis revealed that the combination exhibited synergistic bactericidal activities for tigecycline-resistant isolates during 24 h. The ethidium bromide (EtBr) efflux assay demonstrated that ML-7 could inhibit the functions of efflux pump. Besides, ML-7 disrupted the proton motive force (PMF) *via* increasing ΔpH, which in turn lead to the inhibition of the functions of efflux pump, reduction of intracellular ATP levels, as well as accumulation of ROS. All of which promoted the death of bacteria. And further transcriptomic analysis revealed that genes related to the mechanism of ML-7 mainly enriched in ABC transporters. Taken together, these results revealed the potential of ML-7 as a novel tigecycline adjuvant to circumvent tigecycline-resistant *Klebsiella pneumoniae*.

## Introduction

Antimicrobial resistance has become one of the greatest global public health challenges. In 2019, Centers for Disease Control and Prevention (CDC) listed carbapenem-resistant *Enterobacteriaceae* as an urgent threat for the public (Centers for Disease Control and Prevention, 2019). Particularly, infections caused by carbapenem-resistant *Klebsiella pneumoniae* (CRKP) have been an urgent concern because it is associated with high mortality and morbidity (Jin et al., 2021).

Tigecycline, which belongs to a class of glycylcyclines antibiotics, possesses broad-spectrum antibacterial activity and it is regarded as the last resort antibiotic for treating serious infections caused by multidrug-resistant (MDR) Gram-negative bacteria, especially extensively drug-resistant (XDR) *Enterobacteriaceae* (Doi, 2019). Unfortunately, upon increasing clinical usage, tigecycline-resistant *K. pneumoniae* has been discovered in many countries, including China (Xu et al., 2017), Japan (Hayashi et al., 2021), Vietnam (Hirabayashi et al., 2021) and Austria (Hladicz et al., 2017). The resistance mechanism of tigecycline in *K. pneumoniae* is extensively related to the upregulated transcription of acrAB, oqxAB, and macAB efflux pumps, which results from mutations in transcriptional regulator *ramR* and *acrR* (Sheng et al., 2014; Zheng et al., 2018). Mutations in *rpsJ*, which encodes the ribosome S10 protective protein targeted by tetracyclines, are also associated with tigecycline resistance (He et al., 2018). Besides, mutations in *tet*(A), which was another tetracycline efflux pump gene, have also been reported to cause resistance to tigecycline (Yao et al., 2018). What’s worse, the emergence and rapid spread of *tet*(X3/X4), plasmid-mediated high level tigecycline resistant genes (He et al., 2019; Sun et al., 2019), constitutes a more serious threat to the role of tigecycline as the “last-resort” antibiotic. Therefore, there is an urgent need to explore alternative strategies against tigecycline-resistant pathogens.

Tigecycline-based combination therapies with other antibiotics have been widely used over the past years, but the therapeutic efficiency was unsatisfactory (Bassetti et al., 2015). Different from antibiotics, the non-antibiotic adjuvant strategy could maximize the antimicrobial efficacy of existing antibiotics through enhancing the killing effect of antibiotics, which is a promising approach against antimicrobial resistance. Furthermore, the strategy is able to theoretically slow down the process of antibiotic resistance. Up to now, a few studies have been reported on non-antibiotic adjuvants of tigecycline (Liu et al., 2020; Tong et al., 2021). For example, azidothymidine, as an adjuvant of tigecycline, dramatically potentiates tigecycline activity against Tet(X)-mediated resistance of tigecycline to *E.coli* both *in vitro* and *in vivo* through inhibiting DNA synthesis and suppressing Tet(X) resistance enzyme activity (Liu et al., 2020). Recently, Tong et al. showed that the non-steroidal anti-inflammatory drug benzydamine in combination with tigecycline could reverse *temxCD*-*toprJ*-positive *K. pneumoniae* (Tong et al., 2021).

In this study, we collected 11 clinical tigecycline-resistant *K. pneumoniae* for tigecycline adjuvants screening. Before screening, the mutations of relevant resistance genes and regulators were elucidated. Based on these pathogens, we screened a collection of compounds and fortunately found that ML-7 hydrochloride (ML-7) displayed the most potent synergistic activity with tigecycline. The *in vitro* synergistic activity of ML-7 with tigecycline was evaluated and the mechanisms of ML-7 were also explored. This work provides a promising combination strategy to treat infections caused by tigecycline-resistant *K. pneumoniae*.

## Materials and Methods

### Bacterial and Chemical Agents

A total of 11 clinical strains used in this study were listed in **Table 1**. Tigecycline resistant *K. pneumoniae* clinical isolates were obtained from the CAMS Collection Center of Pathogen Microorganisms (CAMS-CCPM-A) in China. Tigecycline standard powder and N-Phenyl-1-naphthylamine (NPN) were purchased from TCI (Shanghai, China). Propidium iodide (PI) and ethidium bromide (EtBr) were purchased from Sangon Biotech (Shanghai) Co., Ltd. (Shanghai, China). Carbonyl cyanide m-chlorophenyl hydrazone (CCCP), 3,3’-dipropylthiadicarbocyanine iodide [DiSC_3_(5)] and BCECF-AM were purchased from Sigma-Aldrich (St Louis, USA). An enhanced ATP Assay Kit and ROS Assay Kit were purchased from Beyotime Biotechnology (Shanghai, China). ML-7 hydrochloride, ML-9, HA-100, Ripasudil hydrochloride dihydrate, Fasudil hydrochloride, Hydroxyfasudil hydrochloride, tavaborole, and tegaserod maleate were purchased from Target Molecule Company (Boston, USA).

**Table 1:** Multilocus sequence typing (MLST) and MICs of representative antibiotics for the study strains.

Strains	ST	Breakpoints (S-R)	TGC*	FEP	ATM	IPM	AMK	TOB	CIP	LVX	NIT
			2-8	2-16	4-16	1-4	16-64	4-16	1-4	0.5-2	32-128
*K.pneumoniae* 14-R75	11	MIC (μg/mL)	32	≥64	≥64	≥16	≥64	≥16	≥4	≥8	≥512
*K.pneumoniae* 14-R78	1414		256	≥64	≥64	≥16	32	≥16	2	1	≥512
*K.pneumoniae* 14-R71	11		64	≥64	≥64	≥16	≥64	≥16	≥4	≥8	≥512
*K.pneumoniae* 14-R72	11		16	≥64	≥64	≥16	≥64	≥16	≥4	≥8	≥512
*K.pneumoniae* 14-R70	11		128	≥64	≥64	≥16	≥64	≥16	≥4	≥8	≥512
*K.pneumoniae* 14-R74	NA^a^		32	≥64	≥64	≥16	16	4	≥4	≥8	≥512
*K.pneumoniae* 14-R52	1035		8	≤1	≥64	≥16	8	2	≤0.25	0.5	≥512
*K.pneumoniae* 17-R20	14		8	≥64	≥64	≥16	≥64	≥16	≥4	≥8	≥512
*K.pneumoniae* 14-R38	1939		32	≤1	32	≥16	8	≥16	≥4	4	≥512
*K.pneumoniae* 17-R108	3160		8	≥64	≥64	8	4	≥16	1	2	≥512
*K.pneumoniae* 17-R39	1697		16	≥64	≥64	8	8	≥16	≥4	≥8	≥512

ST, sequence typing; TGC, tigecycline; FEP, cefepime; ATM, aztreonam; IPM, imipenem; AMK, amikacin; TOB, tobramycin; CIP, ciprofloxacin; LVX, levofloxacin; NIT, nitrofurantoin.

Breakpoints (S-R): susceptible (S) breakpoint to resistance (R) breakpoint, according to CLSI supplement M100 (31 edition).

*TGC breakpoint was interpreted according to the FDA.

NA^a^, not available, this strain should belong to a new sequence type (ST).

### Antimicrobial Susceptibility Testing

MICs of tigecycline were determined by broth microdilution as recommended by the CLSI (Clinical and Laboratory Standards Institute, 2021). Briefly, bacterial cultures were grown overnight and then diluted to a final concentration of approximately 5 × 10^5^ CFU/mL. The experiment was performed on a 96-well plate where the tested agents were 2-fold serially diluted in CAMHB and incubated with equal volumes of bacterial inoculum at 37°C for 18 h. The breakpoint of tigecycline for *Enterobacteriaceae* was interpreted according to the U.S. Food and Drug Administration (FDA) criteria (susceptible, ≤ 2 μg/mL; intermediate, 4 μg/mL; resistant, ≥ 8 μg/mL) (Sun et al., 2019). The experiment was performed in triplicate on different days. The susceptibility to other antibiotics was evaluated using VITEK 2 Compact System (bioMerieux, France).

### Multilocus Sequence Typing

MLST was performed using seven housekeeping genes (*rpoB*, *gapA*, *mdh*, *pgi*, *phoE*, *infB* and *tonB*) of *K. pneumoniae* (**Table S1**). The experiment was conducted following the protocol as the reference described (Diancourt et al., 2005). The sequences of PCR products were determined by Sangon Biotech Company (Shanghai, China). Allele numbers and sequence types were analyzed on the website https://bigsdb.pasteur.fr/klebsiella/klebsiella.html.

### Characterization of Tigecycline Resistance Determinant Genes

Tigecycline resistant *K. pneumoniae* isolates were screened for the tigecycline resistance genes by performing PCR with specific primers (see **Table S3** in the **Supplemental Material**), as previously described (Chiu et al., 2017). The sequences of PCR products were determined by Sangon Biotech Company (Shanghai, China). Mutations were characterized by comparing the sequences with reference strains [*K. pneumoniae* ATCC 700721 for *acrR*, *rpsJ*, *tet*(A), *macB* (NCBI Reference Sequence NC_009648 and ATCC Genome Portal: 700721) and *K. pneumoniae* ATCC 13883 for *ramR* (GeneBank accession number KY465996.1]. The experiment was repeated in triplicate independently.

### Checkerboard Assays

Synergistic activity between compounds and tigecycline, and the fractional inhibitory concentrations (FIC) indices were measured by checkerboard assays (Zhang et al., 2019). Two drugs were mixed in a 96-well plate to create an 8 × 8 matrix followed by the addition of a standard bacterial suspension at 5 x 10^5^ CFU/mL. The experiment was repeated in triplicate independently. After incubated at 37℃ for 18 h, the results were recorded by naked eyes. The FIC index (FICI) was calculated according to the formula as follows:


$$\text{FICI}=\frac{\text{MIC of drug A in combination}}{\text{MIC of drug A alone}}+\frac{\text{MIC of drug B in combination}}{\text{MIC of drug B alone}}$$

The antimicrobial combination was defined as synergistic when the FICI was ≤ 0.5; indifferent when 0.5 < FICI ≤ 4, and antagonistic when the FICI was > 4.

### Growth Curves

Three single colonies of bacteria were picked and inoculated into 3 mL CAMH broth and grown overnight at 37°C with shaking at 200 rpm. The overnight cultures were standardized to match a 0.5 McFarland and followed by 1:100 dilution in CAMH broth medium. Different concentrations of ML-7, 2 μg/mL tigecycline alone and combination were added to a 100-well microplate. The growth curves were recorded for 24 h under the wavelength of 600 nm with an interval of 1 h at 37°C. The experiment was repeated in triplicate independently.

### Time-Kill Curves

Time-kill curves were performed according to the method described with minor modifications (Cebrero-Cangueiro et al., 2018). Experiments were carried out with a starting inoculum of 1×10^6^ CFU/mL. Tubes were incubated at 37°C without shaking, and samples were taken at 0, 2, 4, 6, 8, and 24 h. The experiment was repeated in triplicate independently. Bactericidal activities of single drugs or combination were defined as a decrease ≥ 3 log_10_ CFU/mL from the starting inoculum, bacteriostatic effect was defined as no change respect to the initial bacterial concentration during the 24 h. Synergy was defined as a decrease ≥ 2 log_10_ CFU/mL for the drugs combination compared with the most active single agent.

### Outer Membrane Permeability Assay

Fluorescent probe NPN was used to evaluated outer membrane permeability. Briefly, bacteria were grown to the mid-log phase (OD_600_ = 0.5). The cultures were washed three times and resuspended with 5 mM HEPES containing 20 mM of glucose (pH=7.2). NPN was added with a final concentration of 10 μM to 100 μL the same buffer containing various concentrations of ML-7, tigecycline alone or combination. Then, 100 μL bacterial solution was added. Fluorescence intensity was measured on a microplate reader with the excitation wavelength at 355 nm and the emission wavelength at 420 nm and the absorbance of different groups at 600 nm. The normalized fluorescence intensity is the ratio of fluorescence intensity to OD_600_. The experiment was repeated in triplicate independently.

### Membrane Integrity Assay

Inner membrane integrity was measured by staining cells with PI. Cells were washed and resuspended with 1 × PBS buffer to obtain an OD_600_ = 0.5. The cells were treated with different concentrations of ML-7, tigecycline or combination. After 30 min, PI was added at a final concentration of 5 μg/mL and kept in the dark at 4°C for 30 min. The fluorescence was measured with the excitation wavelength at 515 nm and emission wavelength at 615 nm. The experiment was repeated in triplicate independently.

### Reactive Oxygen Species Assay

The levels of ROS were measured with 2′,7′-dichlorodihydrofluorescein diacetate (DCFH-DA, 10 μM) according to the manufacturer’s instruction (Beyotime, Shanghai, China). Briefly, bacteria were grown overnight to obtain an OD_600_ of 0.5. DCFH-DA was added to a final concentration of 10 μM and the mixture was incubated at 37°C for 30 min. After that, 180 μL of probe-labeled bacterial cells were added to a 96-well plate containing 20 μL drug liquids. After incubation for another 30 min, fluorescence intensity was immediately measured with the excitation wavelength at 488 nm and the emission wavelength at 525 nm. The experiment was repeated in triplicate independently.

### Ethidium Bromide Efflux Assay

To assess the effect of ML-7 on the inhibition of multidrug efflux pump, an ethidium bromide (EtBr) efflux assay was performed based on a previous study (Maisuria et al., 2015). Briefly, bacterial culture was grown to an OD_600_ ≈ 0.5. Cells were co-incubated with 5 μM EtBr and ML-7 (32-128 μg/mL) or tigecycline (2 μg/mL) alone or combination, or known efflux pump inhibitor CCCP (100 μM) at 37°C for 1 h. After being centrifuged at 5000 g at 4°C g for 10 min, the pellets were collected and resuspended in fresh MH broth. Subsequently, EtBr efflux from the cells was monitored with the excitation wavelength at 530 nm and emission wavelength at 600 nm. The experiment was repeated in triplicate independently.

### ΔpH Measurement

Intracellular pH measurement was conducted as previously described (Song et al., 2021). Briefly, *K. pneumoniae* 14-R75 was grown to an OD_600_ of 0.5 and then washed with 5 mM HEPES containing 5 mM glucose. Cells were treated with 10 μM BCECF-AM for 30 minutes at 37°C. Then tested solution was added and incubated for 30 minutes and the fluorescence value was measured by a Microplate reader (Perkin Elmer), with the excitation wavelength at 488 nm and emission wavelength at 535 nm. The experiment was repeated in triplicate independently.

### Transmembrane Potential Assay

The transmembrane potential was determined as previously with the fluorescent probe DiSC_3_(5) (Ejim et al., 2011). In this assay, mid-log phase cells were pelleted at 5000 × g for 10 min and resuspended in 50 mM HEPES with 20 mM glucose, pH 7.2. Cells loaded with 1 μM DiSC_3_(5) dye were monitored for 30 min for fluorescence to stabilize. After adding tigecycline and/or ML-7, a time-course acquisition was performed immediately at 620 nm excitation and 670 nm emission wavelengths for 30 min.

### ATP Determination

ATP luminescence assay was performed as previously described with slight modifications (Song et al., 2020). Intracellular ATP levels of *K. pneumoniae* 14-R75 were determined using an Enhanced ATP Assay Kit according to the instructions. Luminescence was measured using a Microplate reader (PerkinElmer). The experiment was repeated in triplicate independently.

### Transcriptomic Analysis

Cells from an overnight culture were diluted and incubated to OD_600_ of 0.5. Tigecycline and ML-7 alone or in combination were added and incubated for 4 h. Total RNA was extracted using RNAprep pure Bacteria Kit (TIANGEN, Beijing, China) according to the manufacturer’s instructions. RNA was quantified using NanoDrop spectrophotometers (Thermo Fisher Scientific). Qualified RNA was sequenced by the Illumina HiSeq platform at GENEWIZ Company (Suzhou, China), with a paired-end mode of 2 × 150 bp. The RNA-Seq raw data was filtrated and employed to assemble the bacterial transcriptome using Cutadapt software and RNA-Seq reads were mapped using the bowtie2 software against the genome of *K. pneumoniae* 14-R75. The experiment was designed with three biological replicates.

### Statistical Analysis

All data were presented as the means ± SEM and transcriptomic analysis was presented as means. Experiment results were statistically analyzed with one-way ANOVA and unpaired Student’s t-test by GraphPad Prism version 8.0 software. For sequence alignment of tigecycline-resistant genes, the data was analyzed using SnapGene software.

## Results

### The Clinical Tigecycline-Resistant *K. pneumoniae* Were Multidrug-Resistant and Multisequence Type

We collected 11 clinical tigecycline-resistant *K. pneumoniae* and assessed the susceptibility of tigecycline and eight other antibiotics including aztreonam, imipenem, nitrofurantoin, cefepime, amikacin, tobramycin, ciprofloxacin, and levofloxacin *via* the determination of minimal inhibitory concentrations (MICs). The results were shown in **Table 1**. The tigecycline MIC of the strains ranges from 8 µg/mL to 256 µg/mL. All isolates displayed multidrug resistant phenotypes, and 6 out of 11 isolates were resistant to all of the antibiotics. Among the 11 isolates, all of them were resistant to aztreonam, imipenem, and nitrofurantoin. Meanwhile, cefepime, amikacin, tobramycin, ciprofloxacin, and levofloxacin resistance were existed as well, in most of them.

Further characterization of the isolates was carried out by using MLST. Results showed that seven different sequence types ST 11, 14, 1035, 1414, 1697, 1939 and 3160 were identified among 11 isolates (**Table 1** and **Table S2**).

### Identification of Tigecycline Resistance Mutated Genes in Tigecycline-Resistant *K. pneumoniae* Clinical Isolates

In *K. pneumoniae*, tigecycline resistance is extensively associated with the overexpression of chromosomal resistance nodulation-division type (RND-type) efflux pumps, such as AcrAB and OqxAB (Yaghoubi et al., 2021), which is commonly caused by the mutation of *ramR* and *acrR*. In addition, *tet*(X), *rpsJ*, and *tet*(A) have also been reported to be associated with tigecycline resistance (Yang et al., 2004; Chiu et al., 2017). To determine tigecycline-resistant genes of the 11 isolates, PCR and sequencing of *ramR*, *acrR*, *rpsJ*, *tet(A)* and *macB* using designed full-length primers (**Table S3**) were conducted. The results were presented in **Table 2**. 44% isolates showed base deletion at 368 in *ramR*. For *acrR* and *macB*, there were many nucleotide changes in comparison to the sequence of the reference strain. Among all of the isolates, no mutations of *rpsJ* gene were detected. Besides, the *tet*(X) gene was not found in all isolates (data not shown). Therefore, mutations in *ramR*, *acrR* and *macB* exhibited high prevalence among these isolates.

**Table 2:** Mutations of tigecycline resistance genes in the tigecycline-resistant clinical isolates.

Isolates	*ramR*	*acrR*	*rpsJ*	*tet*(A)	*macB*
*K.pneumoniae* 14-R75	+ (366A367)	+	+	+	+ (S209T, T347S, Δ718, 766C767, 775G776, 778T779)
*K.pneumoniae* 14-R78	+ (Δ368, 372CC373)	+	+	+	+ (S209T, T347S, 747C748, 750C751, 759C760, 765G766, 768T769)
*K.pneumoniae* 14-R71	+ (Δ368, 372CA373)	+	+	+	+ (S209T, T347S, V388L, 582G583, Δ718, 723G724, V769D)
*K.pneumoniae* 14-R72	+ (D367S, A370P)	+	+	+	+ (S209T, T347S, 582G583)
*K.pneumoniae* 14-R74	–	+ (R29L, A59D, T108A, 192T193, I223V, I265V, V286I, T304V)	+	–	–
*K.pneumoniae* 14-R70	–	–	+	+	+ (S209T, T347S, 727T728, 765G766, 790G791)
*K.pneumoniae* 14-R52	+ (368G369)	+	+	+	+ (S209T, T347S)
*K.pneumoniae* 17-R20	+ (Δ368, 372CC373)	+	+	+	+ (S209T, T347S, 555G556, 597T598, insertion 573-574)
*K.pneumoniae* 14-R38	+ (Q304stop)	+ (139G140)	+	+	+ (S209T, T347S, A575V, Δ692,718)
*K.pneumoniae* 17-R108	+ (Δ368, 372CC373)	+	+	+	+ (S209T, T347S, Δ692,718,730, 735, 769G770)
*K.pneumoniae* 17-R39	+ (H350N, 372C373)	+ (Y341F, V493I, S643P)	+	+ (F37L, E591D, Q1178R)	+ (S209T, T347S, V389A, E408D, 582G583, Δ718,747)

Δ, deletion; +, presence of PCR product and no mutations were identified; -, absence of PCR product; bp, base pair.

### ML-7 Is a Potent Adjuvant of Tigecycline

**Figure 1:**
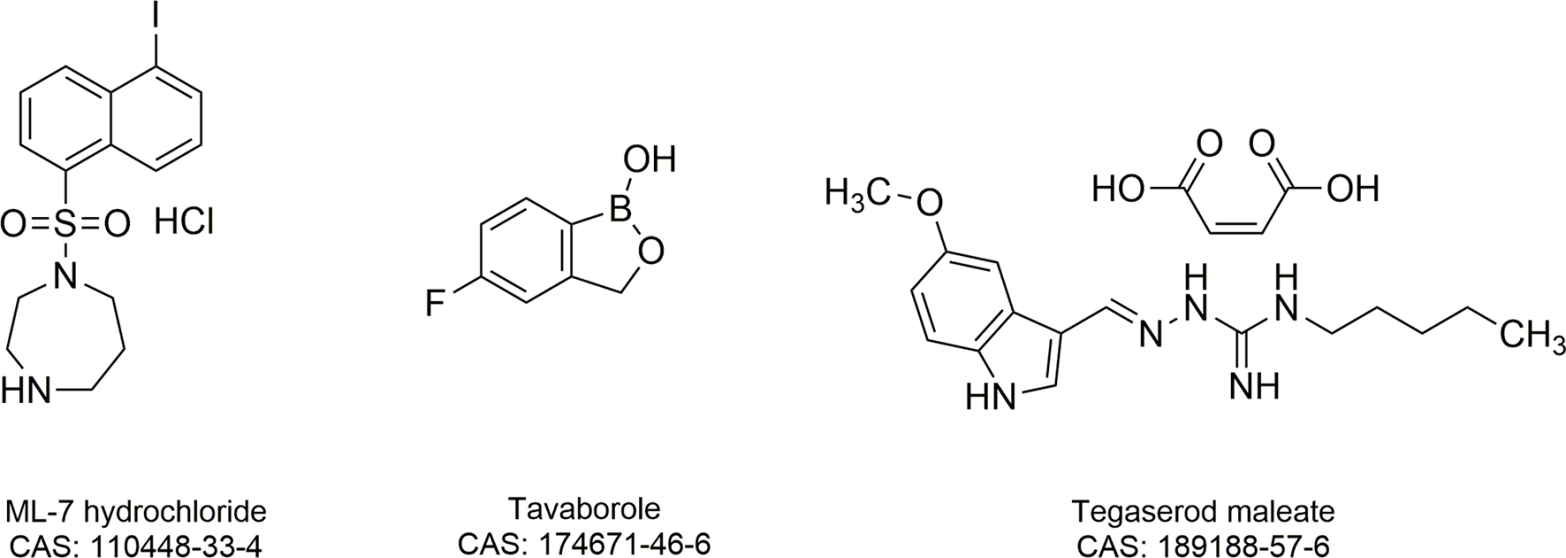
Chemical structures and Chemical Abstracts Service (CAS) registry numbers of ML-7 hydrochloride, tavaborole and tegaserod maleate.

**Figure 2:**
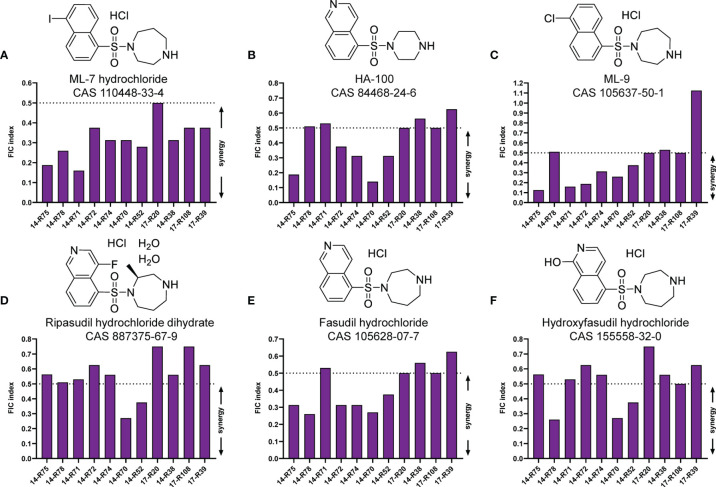
FIC indices of the combination between tigecycline and each of ML-7 analogs against 11 tigecycline-resistant *K. pneumoniae* isolates. **(A)** ML-7 hydrochloride. **(B)** HA-100. **(C)** ML-9. **(D)** Ripasudil hydrochloride dihydrate. **(E)** Fasudil hydrochloride. **(F)** Hydroxyfasudil hydrochloride. Synergy is defined as a FIC index ≤ 0.5.

To find potential tigecycline adjuvants, we performed initial screening among 2711 compounds at the concentration of 64 μg/mL combined with tigecycline at the concentration of 2 μg/mL using *K. pneumoniae* 17-R20. As a result, we found that three compounds, which were ML-7, tavaborole and tegaserod maleate (**Figure 1**), can significantly enhance the susceptibility of *K. pneumoniae* 17-R20 to tigecycline. Checkerboard assay was used to further evaluate the synergistic activity between tigecycline and three compounds against all tigecycline-resistant clinical isolates. As shown in **Table 3**, the combination of ML-7 with tigecycline showed synergistic activity with FICI ≤ 0.5; while both tavaborole and tegaserod maleate displayed selective synergy with tigecycline against 73% tigecycline-resistant isolates. Of particular, we observed that ML-7 exerts the best synergistic effect with tigecycline. Accordingly, we picked up five analogs of ML-7 from the commercial compound library and investigated their synergy effects combined with tigecycline by using checkerboard assay (**Figure 2**). As shown in **Figure 2** and **Table S4**, all analogs exhibited selective synergistic activity with tigecycline and none of them were as good as ML-7. Therefore, the combination of ML-7 with tigecycline was selected for further investigation.

**Table 3:** MICs of tigecycline combination with ML-7 hydrochloride, tavaborole and tegaserod maleate for all tested isolates.

Strains	Drugs	MIC		FICI	Drugs	MIC		FICI	Drugs	MIC		FICI
		Alone	COMB			Alone	COMB			Alone	COMB	
*K.pneumoniae* 14-R75	TGC	32	2 (**16**)	0.1875	TGC	32	8 **(4)**	0.75	TGC	32	4 **(8)**	0.375
	ML-7	512	64		tavaborole	64	32	tegaserod maleate	32	8
*K.pneumoniae* 14-R78	TGC	256	2 (**128**)	0.26	TGC	256	0.12 (**2048**)	0.13	TGC	256	0.12 (**2048**)	0.25
	ML-7	512	128		tavaborole	512	64	tegaserod maleate	128	32
*K.pneumoniae* 14-R71	TGC	64	2 (**32**)	0.16	TGC	64	8 **(8)**	0.375	TGC	64	4 **(16)**	0.31
	ML-7	512	64		tavaborole	128	32	tegaserod maleate	32	8
*K.pneumoniae* 14-R72	TGC	16	2 (**8**)	0.375	TGC	16	0.12 **(128)**	1.01	TGC	16	2 **(8)**	0.38
	ML-7	256	64		tavaborole	64	64	tegaserod maleate	32	8
*K.pneumoniae* 14-R74	TGC	32	2 (**16**)	0.3125	TGC	32	8 **(4)**	0.27	TGC	32	8 **(4)**	0.5
	ML-7	256	64		tavaborole	64	1	tegaserod maleate	32	8
*K.pneumoniae* 14-R70	TGC	128	2 **(64)**	0.3125	TGC	128	0.12 **(1024)**	0.06	TGC	128	4 (**32**)	0.09
	ML-7	128	32		tavaborole	512	32	tegaserod maleate	16	1
*K.pneumoniae* 14-R52	TGC	8	2 (**4**)	0.28	TGC	8	1 (**8**)	0.25	TGC	8	0.5 (**16**)	0.56
	ML-7	128	4		tavaborole	256	32	tegaserod maleate	32	16
*K.pneumoniae* 17-R20	TGC	8	2 (**4**)	0.5	TGC	8	2 (**4**)	0.5	TGC	8	0.12 (**64**)	0.265
	ML-7	256	64		tavaborole	64	16	tegaserod maleate	64	16
*K.pneumoniae* 14-R38	TGC	32	2 (**16**)	0.3125	TGC	32	2 (**16**)	0.19	TGC	32	4 (**8**)	0.375
	ML-7	512	128		tavaborole	256	32	tegaserod maleate	32	8
*K.pneumoniae* 17-R108	TGC	8	2 (**4**)	0.375	TGC	8	0.25 (**32**)	0.28	TGC	8	0.5 (**16**)	0.56
	ML-7	256	32		tavaborole	128	32	tegaserod maleate	32	16
*K.pneumoniae* 17-R39	TGC	16	2 (**8**)	0.375	TGC	16	0.12 (**128**)	0.51	TGC	16	0.25 (**64**)	0.52
	ML-7	512	128		tavaborole	64	32	tegaserod maleate	32	16

TGC, tigecycline; ML-7, ML-7 hydrochloride; COMB, combination; FICI, fractional inhibitory concentration index. Synergy was defined as FICI ≤ 0.5, indifference as 0.5＜FICI ≤ 4, and antagonism as FICI > 4. The bold values mean the fold of reduction (which is a acquired by: Alone/COMB).

**Figure 3:**
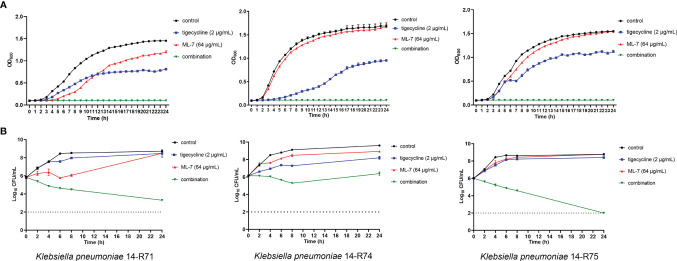
Growth curves **(A)** and time-kill curves **(B)** of ML-7 and tigecycline alone or in combination against *K. pneumoniae* 14-R71, 14-R74 and 14-R75. ML-7, ML-7 hydrochloride.

Growth curves and time-kill curves with ML-7 and tigecycline alone or in combination were presented in **Figure 3**. Data from representative strains was shown to display the synergistic activities. The results from growth curves showed that 64 μg/mL ML-7 and 2 μg/mL tigecycline alone could inhibit the growth of bacteria to a certain extent; whereas the combined incubation could completely inhibit the growth of bacteria (**Figure 3A**). Time-kill curves of three tigecycline-resistant *K. pneumoniae* strains 14-R71, 14-R74, and 14-R75 were revealed in **Figure 3B**. There was no obvious influence on strains treated with tigecycline or ML-7 alone during 24 h incubation. The combination of ML-7 and tigecycline exhibited both synergistic and constant bactericidal activities for 14-R71 and 14-R75 with a ~5-6 log_10_ CFU/mL reduction. As for 14-R74, synergistic bacteriostatic activity was observed over 24 h for the combination of ML-7 and tigecycline, compared with ML-7 or tigecycline alone. These findings suggested that ML-7 could enhance the killing activity of tigecycline.

### Synergistic Mechanism of ML-7 in Combination With Tigecycline

**Figure 4:**
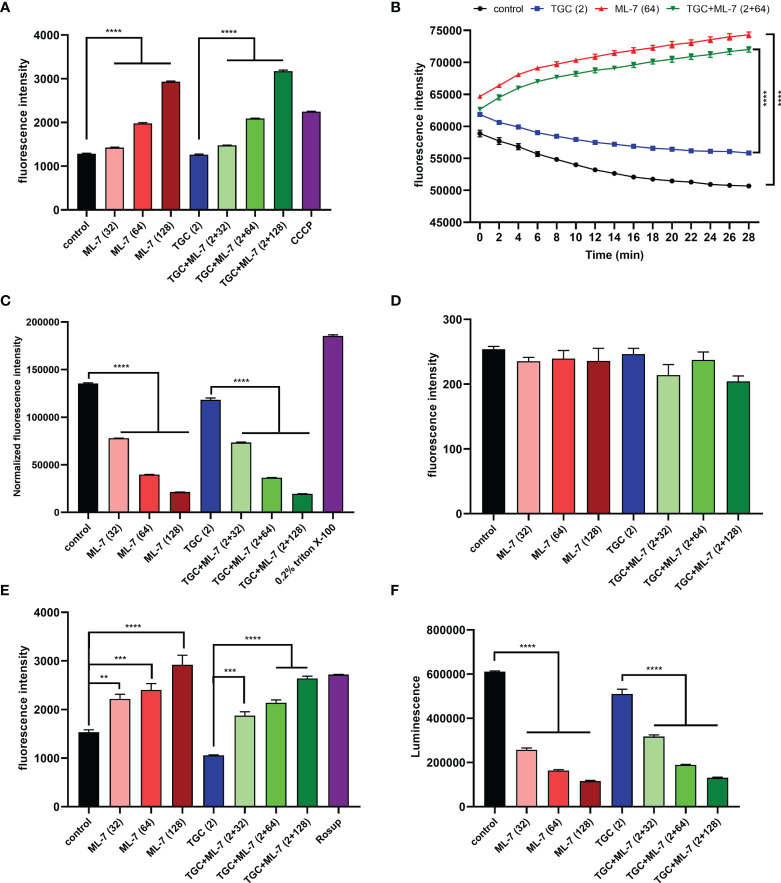
Mechanism of ML-7 in combination with tigecycline. **(A)** Inhibition of efflux pump by ML-7. Efflux pump inhibitor, CCCP, was recognized as the positive control. **(B)** Increased ΔpH by ML-7 in treated cells. **(C)** Permeability of the outer membrane probed with NPN. 0.2% Triton X-100 was recognized as the positive control. The normalized fluorescence intensity is the ratio of fluorescence intensity to OD_600_. **(D)** Permeability of the inner membrane probed with PI. **(E)** Total ROS accumulation in *K*. *pneumoniae* 14-R75 treated with ML-7 alone or in combination with tigecycline. Rosup was recognized as the positive control. **(F)** Decreased intracellular ATP levels after the treatment of ML-7. All experiments were performed as three biologically independent experiments, data presented as mean ± SEM, n = 3 (for F, two biologically independent experiments, n = 2). *P*-values in **(A, C–F)** were calculated using non-parametric one-way ANOVA. *P*-values in **(B)** were calculated using unpaired Student’s t-test. ^**^ *P* < 0.01, ^***^ *P* < 0.001, ^****^ *P* < 0.0001. TGC, tigecycline; ML-7, ML-7 hydrochloride; COMB, combination of tigecycline with tigecycline. Units, μg/mL.

Previous studies have been shown that overexpression of RND-efflux pumps plays an important role in tigecycline resistance in *K. pneumoniae* (Lv et al., 2020). To assess if ML-7 was capable of inhibiting the efflux pump, we explored the effect of ML-7 on the functions of efflux pump by using ethidium bromide (EtBr) as a fluorescent probe. As shown in **Figure 4A**, the application of ML-7 alone caused significant increase of fluorescence intensity compared with the control group. When ML-7 was combined with tigecycline, the fluorescence intensity also exerted a dose-dependent increase compared with tigecycline alone. These results indicated that ML-7 showed the synergistic effect with tigecycline by inhibiting drug resistance efflux pump. Furthermore, we evaluated the ΔpH, a key component of the proton motive force (PMF) (Farha et al., 2013), by using a pH indicator BCECF-AM. Compared with control and tigecycline alone, ML-7 significantly increased the level of ΔpH, implying the disruption of PMF in bacteria (**Figure 4B**). Transmembrane potential (Δψ) was another important parameter of PMF. Membrane potential-sensitive dye DiSC_3_(5) was used to measure transmembrane potential of bacteria. If the bacterial membrane is in its native hyperpolarized state, the cationic dye accumulates in the membrane and its fluorescence emission is self-quenched. However, if the membrane is disrupted, the membrane potential will be dissipated and the dye will be released into the medium, causing an increase in fluorescence (Farha et al., 2013). Upon dissipation in ΔpH, bacterial cells will compensate by a counteracting increase in Δψ in order to maintain a constant PMF. So DiSC_3_(5) can also inform on disruption in ΔpH through observed decreases in fluorescence. As expected, we observed that ML-7 led to a significant decrease in fluorescence in the presence or absence of tigecycline, which further indicating ML-7 selectively disrupt ΔpH (**Figure S1**).

Next, we characterized the impact of ML-7 on the permeability of outer membrane by using hydrophobic fluorophore 1-N-phenylnapthylamine (NPN). Interestingly, we observed that the fluorescence intensity markedly decreased in a dose-dependent manner after the addition of ML-7 (**Figure 4C**). We further investigated the effect of ML-7 on the integrity of inner membrane by using a fluorescent probe propidium iodide (PI). However, no significant increase of fluorescence was observed when addition of ML-7 in the presence or absence of tigecycline (**Figure 4D**). These results demonstrated that ML-7 decreased the outer membrane permeability; while had no impact on the permeability of inner membrane.

Subsequently, we measured the sequential responses to decipher the synergy between ML-7 and tigecycline. Previous studies had reported that the PMF is also related to the overproduction of reactive oxygen species (ROS) (Dupuy et al., 2009). To examine whether ML-7 treatment could result in an increased production of ROS, we tested levels of ROS in cells treated with ML-7 and/or tigecycline. As expected, we found that ML-7 could trigger the accumulation of intracellular ROS no matter tigecycline presence or not (**Figure 4E**). Besides, we observed that the intracellular levels of ATP significantly decreased in a dose-dependent manner in cells treated with ML-7 (**Figure 4F**).

**Figure 5:**
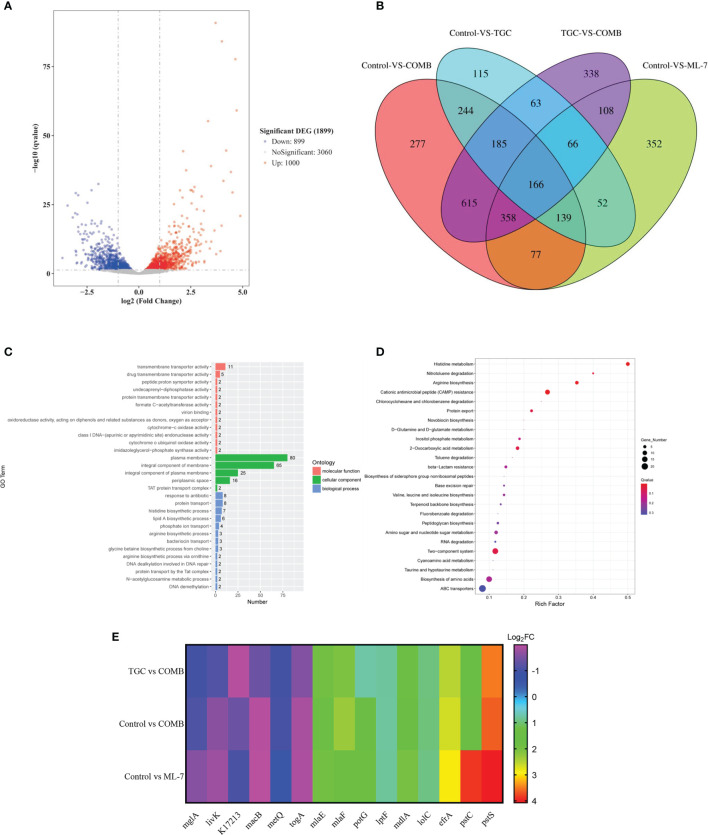
Transcriptome analysis of *K. pneumoniae* 14-R75 after exposure to tigecycline or ML-7 alone and in combination for 4 h. **(A)** Volcano plots of genes after treatment by tigecycline plus ML-7 compared to tigecycline treated cells. **(B)** Venngram of genes identified in four groups. **(C, D)** Functional analysis of 358 targeted genes. **(E)** Heatmap of selected genes related to ABC transporters. Data were presented as means of three biological replicates. TGC, tigecycline alone; COMB, the combination of tigecycline and ML-7; ML-7, ML-7 hydrochloride; Log_2_FC, Log_2_ Fold Change.

To gain insight into the mechanisms of ML-7, we performed transcription analysis of *K. pneumoniae* 14-R75 exposure to tigecycline and ML-7 alone or in combination for 4 h. Compared with tigecycline alone, following treatment with the combination of tigecycline with ML-7, the transcription of 1000 genes were significantly up-regulated and 899 genes were significantly down-regulated (**Figure 5A**). As shown in **Figure 5B**, Venn diagram analysis of four different treated groups was performed. In order to further identify the targeted genes associated with the mechanism of ML-7, we firstly narrowed it down to the overlapping regions between TGC VS COMB and Control VS ML-7. Subsequently, we excluded the genes located in tigecycline treated groups from overlapping regions and finally targeted 358 genes, which were the overlapping regions among TGC VS COMB, Control VS ML-7 and Control VS COMB. GO annotation analysis showed that these genes were mainly correlated with the plasma membrane and their molecular function was mainly transmembrane transporter activity (**Figure 5C**). The results of KEGG pathway analysis demonstrated that these genes were markedly enriched in ABC transporters (**Figure 5D**). ABC transporters were a series of transmembrane transport systems aimed at importing or exporting a wide range of molecules across membranes, including antibiotics, sugars and lipids and so on, such as efflux pumps (Kolich et al., 2020). We selected 15 genes involved in ABC transporters from 358 genes. As shown in **Figure 5E**, we found that 6 genes were down-regulated and 9 genes were up-regulated. The results further verified that ML-7 showed synergistic activity with tigecycline through efflux pumps.

**Figure 6:**
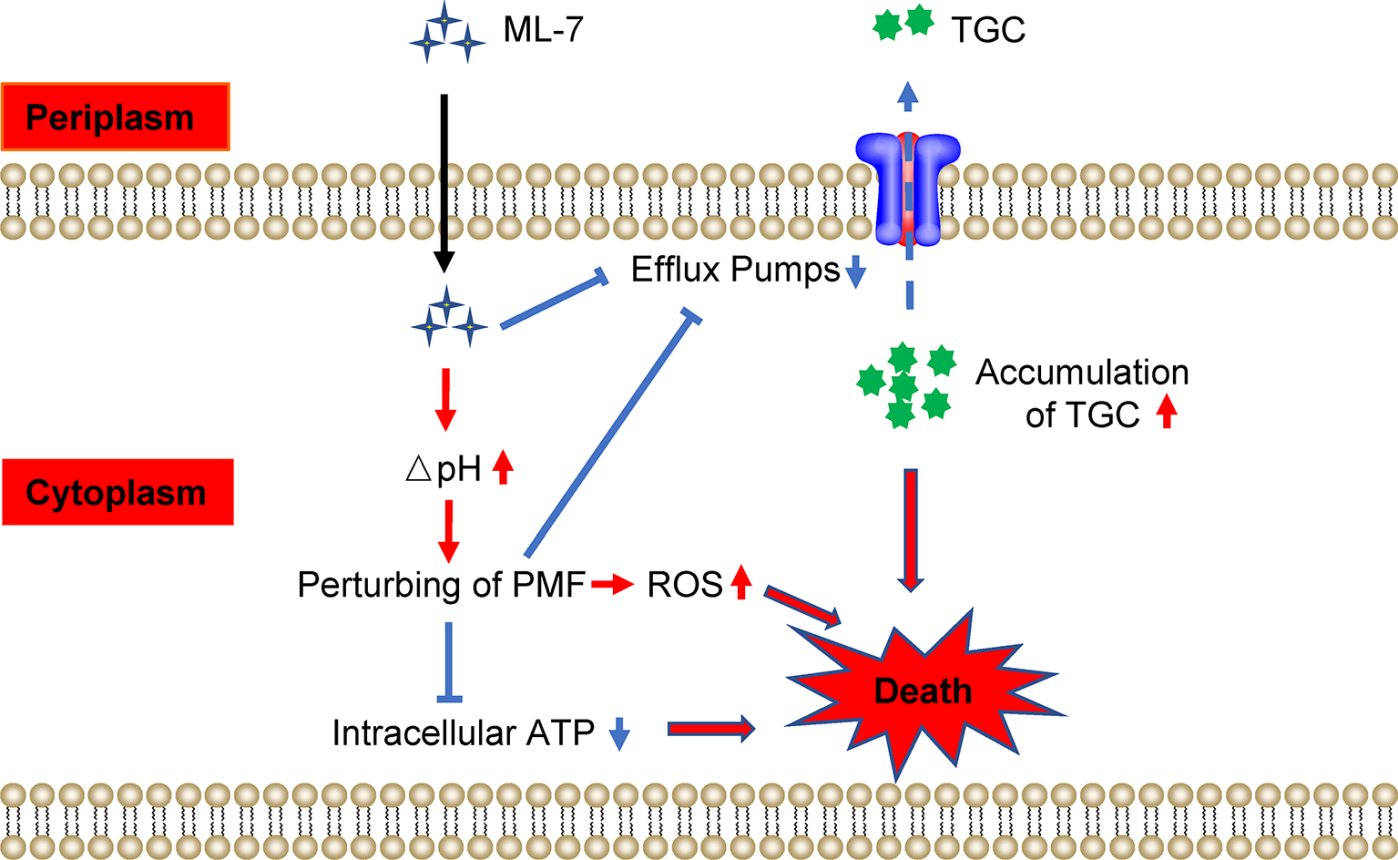
Scheme of the synergy of ML-7 in combination with tigecycline against tigecycline-resistant *K. pneumoniae*. ML-7 enhances tigecycline activity through two pathways: (1) ML-7 promoted intracellular accumulation of tigecycline *via* inhibiting the functions of multidrug efflux pump; (2) ML-7 increased ΔpH and lead to perturb the proton motive force (PMF), which subsequently inhibited the functions of efflux pump, decreased the levels of intracellular ATP and resulted in the accumulation of ROS. All of which promoted the death of bacteria. TGC, tigecycline; ML-7, ML-7 hydrochloride. The red arrows mean promotion; blue arrows mean inhibition.

Collectively, our findings indicated that ML-7 restored the susceptibility of tigecycline-resistant *K. pneumoniae* through two pathways: (1) ML-7 could inhibit the functions of efflux pump and lead to the accumulation of tigecycline in bacteria; (2) ML-7 increased ΔpH and resulted in perturbing the PMF, which subsequently inhibited the functions of efflux pump, decreased the levels of intracellular ATP and resulted in the accumulation of ROS (**Figure 6**).

## Discussion

In recent years, the development and rapid spread of antimicrobial resistance represent a major threat to human health worldwide. Carbapenems have been widely used to against MDR *Enterobacteriaceae*. Unfortunately, with the increased use, carbapenem-resistant *Enterobacteriaceae* has been emerged and spread rapidly, especially in *K. pneumoniae* (Ni et al., 2021). Infections induced by CRKP have been considered a great challenge in clinical practices (Agyeman et al., 2020). Treatment options for CRKP are very limited, and they are generally only susceptible to polymyxins, tigecycline (Durante-Mangoni et al., 2019). However, no matter if it is monotherapy or in combination, the clinical efficacy is not satisfactory. Besides, we found that pan drug-resistant *K. pneumoniae* is increasingly being reported worldwide and is associated with high mortality (Karakonstantis et al., 2020). Therefore, there is an urgent need to explore effective strategies to deal with this challenge. The goal of this study was to characterize the resistance mechanism of 11 *K. pneumoniae* clinical isolates and screen non-antibiotic adjuvants of tigecycline against MDR *K. pneumoniae*.

The tigecycline resistance determinants were detected in 11 *K. pneumoniae* clinical isolates, which exhibited multidrug-resistant phenotype. Mutations of most strains were found in *ramR*, *acrR* and *macB* genes, which were the regulators of the efflux pump. Consistently, previous studies have also demonstrated that the resistance mechanisms of CRKP to tigecycline were associated with the efflux pumps and regulators (Pournaras et al., 2016).

After the screening of in-house compounds library, we discovered that several compounds could exert the synergist effect with tigecycline, which including ML-7, tavaborole, tegaserod maleate and analogs of ML-7. Of particular, ML-7 exerted the greatest synergy with tigecycline and thereby was performed for our further assessment. ML-7 is an inhibitor of myosin light chain kinase (MLCK) (Xiong et al., 2017). It has been found it is beneficial to heart ischemia/reperfusion injury (Lin et al., 2012), inflammatory bowel disease (IBD) (Xiong et al., 2017), and atherosclerosis (Cheng et al., 2015). However, the potential application of ML-7 toward bacteria diseases has not been reported so far. In the present study, we found that ML-7 effectively potentiated the antibacterial activity of tigecycline against tigecycline-resistant *K. pneumoniae*, which belonging to 7 different sequence types, indicating tigecycline MIC reduction induced by ML-7 might be a general rule for different sequence type *K. pneumoniae* isolates.

Efflux pumps play a vital role in the resistance of tigecycline in *K. pneumoniae*. Our results showed that ML-7 was able to undermine the functions of multidrug efflux pump. Meanwhile, tetracycline accumulation is a ΔpH-dependent process (Yamaguchi et al., 1991). The ΔpH was increased in ML-7-treated bacteria, which in turn would enhance the uptake of tigecycline. Consequently, the above together might promote intracellular accumulation of tigecycline.

The unexpected new finding of the present study was that ML-7 decreased permeability of outer membrane in a dose-dependent manner, while no effect on inner permeability. These results implied that the potentiation activity of ML-7 with tigecycline was independent of membrane disruption.

As an electrochemical proton gradient across the cytoplasmic membrane, PMF powers ATP synthesis (Maloney et al., 1974) and RND-type efflux pumps transport (Zgurskaya et al., 2018) in bacteria. ML-7 disrupted the homeostasis of PMF *via* increasing ΔpH, which in turn lead to the reduction of intracellular ATP levels, inhibition of the functions of efflux pump as well as accumulation of ROS, which could potentiate the bactericidal activity of tigecycline and promote the death of bacteria. ROS represents a novel antimicrobial strategy. Oxidative stress, caused by ROS, can damage DNA, RNA, proteins and lipids (Brynildsen et al., 2013), and disturb the cellular oxidative environment and induce cell death (Dharmaraja, 2017). Therapies involving ROS as a mechanism of action are already available in clinical use for wounds (Dryden, 2018). Previous study has been demonstrated that increased ROS production could potentiate killing by antibiotics, including ampicillin, kanamycin, norfloxacin and aminoglycoside (Lam et al., 2020). A common mechanism mainly involved the tricarboxylic acid cycle, a transient depletion of NADH, destabilization of iron-sulfur clusters, and stimulation of the Fenton reaction (Kohanski et al., 2007). Recently, it has been reported that a peptide called SLAP-S25 could trigger the accumulation of ROS in bacteria, and such property had made it be a broad-spectrum antibiotic adjuvant (Song et al., 2020). Consistently, our results revealed that ML-7, as an adjuvant of tigecycline, also promoted the accumulation of ROS.

Analysis of transcriptome revealed that genes associated with the mechanism of ML-7 were mainly involved in ABC transporters. It had been demonstrated that MacAB-TolC pump assembly, as a bacterial ABC drug exporter, was able to extrude antibiotics (Zheng et al., 2018). We found the transcription of *macB* gene, as a component of MacAB-TolC pump, was down-regulated, which lead to the inhibition of the functions of efflux pump. It was consistent with the results from ethidium bromide (EtBr) efflux assay. In order to maintain lipid asymmetry of the outer membrane, LptBFGC, LolCDE and MlaBDEF were required to transport outer membrane components, including LPS, lipoprotein and glycerophospholipids (GPL) from cytoplasm to the outer membrane (Rao et al., 2020). Our results showed that *mlaE*, *mlaF*, *lptF* and *lolC* were significantly up-regulated, which may be the reason for the decreased permeability of outer membrane by ML-7.

In summary, in this study, we characterized the mutations of resistance genes of 11 tigecycline-resistant *K. pneumoniae* and further assessed the *in vitro* synergistic activity of tigecycline combined with ML-7 against tigecycline-resistant *K. pneumoniae*. We conclude that ML-7 has remarkable power as a tigecycline adjuvant. More importantly, our work provides a new scaffold lead compound for tigecycline adjuvant discovery and may have broad prospects in discovering more potential antibiotic adjuvants.

## Data Availability Statement

The datasets presented in this study can be found in online repositories. The names of the repository/repositories and accession number(s) can be found below: Sequence Read Archive (SRA); SRR16962309, SRR16962310, SRR16962311, and SRR16962312.

## Author Contributions

YZ and LiS conceived and designed the study. LiS, XH, XW and TN performed the experiments. LiS analyzed the data and drafted the manuscript. LaS, XL, and YZ revised the manuscript. YZ and XY finalized the manuscript. All authors contributed to the article and approved the submitted version.

## Funding

This research was supported by the National Natural Science Foundation of China (grant number 32141003, 82104248, 82104249 and 81803413), the CAMS Innovation Fund for Medical Sciences (CIFMS) (grant number 2021-1-12M-030, 2021-1-12M-039), the Fundamental Research Funds for the Central Universities (grant number 2021-PT350-001 and 2020-PT310-003), and the National Science and Technology Infrastructure of China (no. National Pathogen Resource Center-NPRC-32).

## Conflict of Interest

The authors declare that the research was conducted in the absence of any commercial or financial relationships that could be construed as a potential conflict of interest.

## Publisher’s Note

All claims expressed in this article are solely those of the authors and do not necessarily represent those of their affiliated organizations, or those of the publisher, the editors and the reviewers. Any product that may be evaluated in this article, or claim that may be made by its manufacturer, is not guaranteed or endorsed by the publisher.
