# Supplementary material for: A Novel Tigecycline Adjuvant ML-7 Reverses the Susceptibility of Tigecycline-Resistant Klebsiella pneumoniae
Source: Front Cell Infect Microbiol. 2022 Jan 5;11:809542. doi: 10.3389/fcimb.2021.809542 (PMC8766836; doi:10.3389/fcimb.2021.809542)
Supplement: Supplementary file 1 [file DataSheet_1.docx]

**Figure S1**


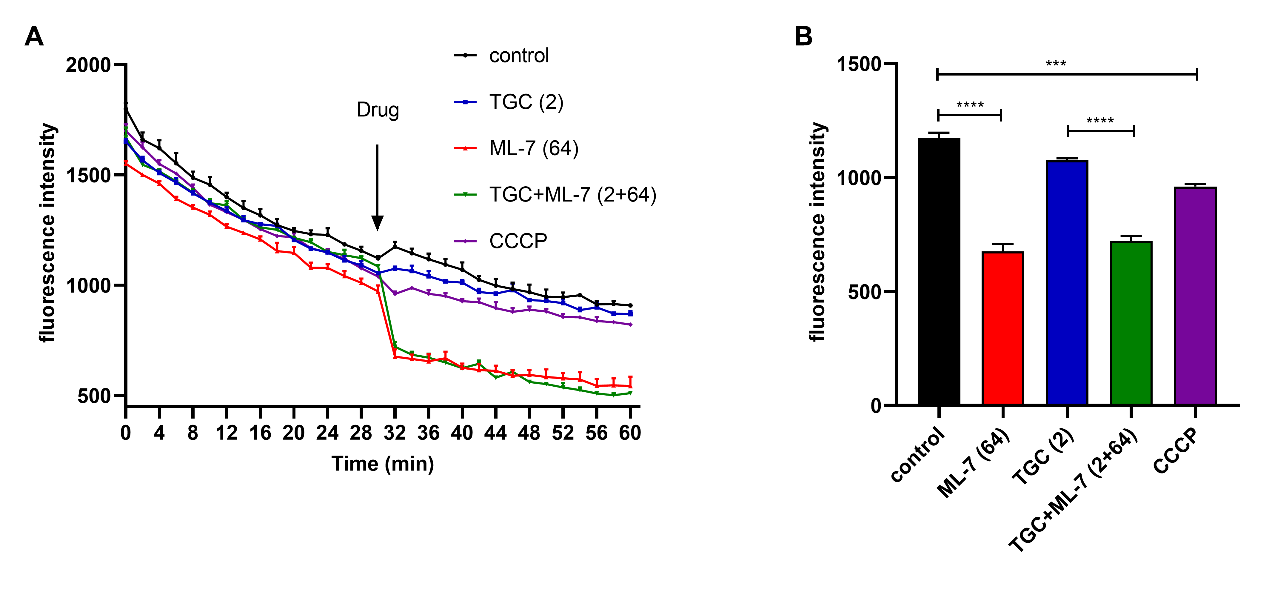


**Figure S1.** ML-7 decreased the fluorescence of DiSC_3_(5). A, DiSC_3_(5) was added followed by self-quenching and stabilization. The various compounds were then injected at 30 min. B, Fluorescence of different groups after addition of ML-7 and tigecycline at 32 min. The experiment was performed as three biologically independent experiments, data presented as mean ± SEM, n = 3. *P*-values were calculated using non-parametric one-way ANOVA. ^***^*P* < 0.001, ^****^*P* < 0.0001. TGC, tigecycline; ML-7, ML-7 hydrochloride; CCCP (15 μM) was recognized as the positive control. Units, μg/mL.

**Table S1** Primers used for MLST of *K. pneumoniae* isolates.

| Gene name | Sequence（5’-3’） | |  |
| --- | --- | --- | --- |
| *rpoB*-F | | GTTTTCCCAGTCACGACGTTGTAGGCGAAATGGCWGAGAACCA | |
| *rpoB*-R | | TTGTGAGCGGATAACAATTTCGAGTCTTCGAAGTTGTAACC | |
| *gapA*-F | | GTTTTCCCAGTCACGACGTTGTATGAAATATGACTCCACTCACGG | |
| *gapA*-R | | TTGTGAGCGGATAACAATTTCCTTCAGAAGCGGCTTTGATGGCTT | |
| *mdh*-F | | GTTTTCCCAGTCACGACGTTGTA CCCAACTCGCTTCAGGTTCAG | |
| *mdh*-R | | TTGTGAGCGGATAACAATTTCCCGTTTTTCCCCAGCAGCAG | |
| *pgi*-F | | GTTTTCCCAGTCACGACGTTGTAGAGAAAAACCTGCCTGTACTGCTGGC | |
| *pgi*-R | | TTGTGAGCGGATAACAATTTCCGCGCCACGCTTTATAGCGGTTAAT | |
| *phoE*-F | | GTTTTCCCAGTCACGACGTTGTAACCTACCGCAACACCGACTTCTTCGG | |
| *phoE* -R | | TTGTGAGCGGATAACAATTTCTGATCAGAACTGGTAGGTGAT | |
| *infB* -F | | GTTTTCCCAGTCACGACGTTGTA**CTCGCTGCTGGACTATATTCG** | |
| *infB*-R | | TTGTGAGCGGATAACAATTTC CGCTTTCAGCTCAAGAACTTC | |
| *tonB-*F | | GTTTTCCCAGTCACGACGTTGTACTTTATACCTCGGTACATCAGGTT | |
| *tonB-*R | | TTGTGAGCGGATAACAATTTCATTCGCCGGCTGRGCRGAGAG | |

**Table S2** Multilocus sequence typing (MLST) and genotypes of *K. pneumoniae* isolates.

| Strains | Year | Sample | MLST | | | | | | | ST | Genotype |
| --- | --- | --- | --- | --- | --- | --- | --- | --- | --- | --- | --- |
|  |  |  | *rpoB* | *gapA* | *mdh* | *pgi* | *phoE* | *infB* | *tonB* |  |  |
| 14-R75 | 2014 | Human | 1 | 3 | 1 | 1 | 1 | 3 | 4 | 11 | *tetA*(48), *mcr*-4, *AIM*-1, *SHV*-64 |
| 14-R78 | 2014 | Human | 96 | 2 | 20 | 1 | 10 | 1 | 38 | 1414 | - |
| 14-R71 | 2014 | Human | 1 | 3 | 1 | 1 | 1 | 3 | 4 | 11 | *tetA*(48), *SHV*-11, |
| 14-R72 | 2014 | Human | 1 | 3 | 1 | 1 | 1 | 3 | 4 | 11 | - |
| 14-R74 | 2014 | Human | 165 | 168 | 426 | 197 | 329 | 196 | 790 | NA^a^ | *tetA*(48), *mcr*-4, *AIM*-1, |
| 14-R70 | 2014 | Human | 1 | 3 | 1 | 1 | 1 | 3 | 4 | 11 | - |
| 14-R52 | 2014 | Human sputum | 1 | 4 | 1 | 26 | 1 | 1 | 4 | 1035 | - |
| 17-R20 | 2017 | Human blood | 1 | 1 | 1 | 1 | 1 | 6 | 1 | 14 | *KPC*-2 |
| 14-R38 | 2014 | Human sputum | 15 | 2 | 174 | 13 | 16 | 1 | 4 | 1939 | - |
| 17-R108 | 2017 | Human bile | 25 | 2 | 1 | 97 | 3 | 1 | 127 | 3160 | - |
| 17-R39 | 2017 | Human abscess | 13 | 18 | 18 | 16 | 25 | 1 | 165 | 1697 | *IMP*-26 |

NA^a^, not available, this strain should belong to a new sequence type (ST).

**Table S3** The sequences of primers used in this study.

| Gene name | Sequence（5’-3’） | |  |
| --- | --- | --- | --- |
| *ramR*-F | | GTGGCTCGTCCAAAGAGTGA | |
| *ramR*-R | | TCATTTGGCGTCCGCCTCAT | |
| *acrR*-F | | ATGTAAACCTCGAGTGTCCA | |
| *acrR*-R | | TCGTCGCAAGCGCGCAGAAT | |
| *rpsJ*-F | | GTCGCGCCAGAATTTGCGTAT | |
| *rpsJ*-R | | TGTTTCAACCTCTCAATCGC | |
| *tet*(A)-F | | GCATAAAGCGCTATGAGC | |
| *tet*(A)-R | | TACAGCGAAACGTTTTACCA | |
| *macB*-F1 | | AACGTGATTACTATCCCGCT | |
| *macB*-R1 | | CTCTTTTGCTTCATTGTGTACATCC | |
| *macB*-F2 | | GATATTCGCGCCATCGGC | |
| *macB*-R2 | | GCAGGCGCAGGCTTTTTG | |

**Table S4** MICs of tigecycline combination with five analogs of ML-7 hydrochloride for all tested *K. pneumoniae* isolates.

| Strains | Drugs | MIC | | FICI | Drugs | MIC | | FICI | Drugs | MIC | | FICI | Drugs | MIC | | FICI | Drugs | MIC | | FICI |
| --- | --- | --- | --- | --- | --- | --- | --- | --- | --- | --- | --- | --- | --- | --- | --- | --- | --- | --- | --- | --- |
|  |  | Alone | COMB |  |  | Alone | COMB |  |  | Alone | COMB |  |  | Alone | COMB |  |  | Alone | COMB |  |
| 14-R75 | TGC | 32 | 2(**16**) | 0.1875 | TGC | 32 | 2 **(16)** | 0.125 | TGC | 32 | 2 **(16)** | 0.5625 | TGC | 32 | 2(**16**) | 0.3125 | TGC | 32 | 2 **(16)** | 0.5625 |
|  | HA-100 | 1024 | 256 |  | ML-9 | 256 | 16 |  | Ripasudil | 1024 | 512 |  | Fasudil | 1024 | 256 |  | Hydroxyfasudil | 1024 | 512 |  |
| 14-R78 | TGC | 256 | 2(**128**) | 0.51 | TGC | 256 | 2 (**128**) | 0.51 | TGC | 256 | 2(**128**) | 0.51 | TGC | 256 | 2(**128**) | 0.26 | TGC | 256 | 2 (**128**) | 0.26 |
|  | HA-100 | 1024 | 512 |  | ML-9 | 512 | 256 |  | Ripasudil | 1024 | 512 |  | Fasudil | 1024 | 256 |  | Hydroxyfasudil | 1024 | 256 |  |
| 14-R71 | TGC | 64 | 2(**32**) | 0.53 | TGC | 64 | 2 **(32)** | 0.16 | TGC | 64 | 2 **(32)** | 0.53 | TGC | 64 | 2(**32**) | 0.53 | TGC | 64 | 2**(32)** | 0.53 |
|  | HA-100 | 1024 | 512 |  | ML-9 | 256 | 32 |  | Ripasudil | 1024 | 512 |  | Fasudil | 1024 | 512 |  | Hydroxyfasudil | 1024 | 512 |  |
| 14-R72 | TGC | 16 | 2 (**8**) | 0.375 | TGC | 16 | 2 **(8)** | 0.1875 | TGC | 16 | 2 **(8)** | 0.625 | TGC | 16 | 1 (**16**) | 0.3125 | TGC | 16 | 2**(8)** | 0.625 |
|  | HA-100 | 1024 | 256 |  | ML-9 | 256 | 16 |  | Ripasudil | 1024 | 512 |  | Fasudil | 1024 | 256 |  | Hydroxyfasudil | 1024 | 512 |  |
| 14-R74 | TGC | 32 | 2(**16**) | 0.3125 | TGC | 32 | 2 **(16)** | 0.3125 | TGC | 32 | 2 **(16)** | 0.56 | TGC | 32 | 2(**16**) | 0.3125 | TGC | 32 | 2 **(16)** | 0.56 |
|  | HA-100 | 1024 | 256 |  | ML-9 | 512 | 128 |  | Ripasudil | 1024 | 512 |  | Fasudil | 1024 | 256 |  | Hydroxyfasudil | 1024 | 512 |  |
| 14-R70 | TGC | 128 | 2 **(64)** | 0.14 | TGC | 128 | 1**(128)** | 0.26 | TGC | 128 | 2 (**64**) | 0.27 | TGC | 128 | 2 **(64)** | 0.27 | TGC | 128 | 2**(64)** | 0.27 |
|  | HA-100 | 1024 | 128 |  | ML-9 | 256 | 64 |  | Ripasudil | 1024 | 256 |  | Fasudil | 1024 | 256 |  | Hydroxyfasudil | 1024 | 256 |  |
| 14-R52 | TGC | 8 | 2(**4**) | 0.3125 | TGC | 8 | 2 (**4**) | 0.375 | TGC | 8 | 2 (**4**) | 0.375 | TGC | 8 | 2(**4**) | 0.375 | TGC | 8 | 2 (**4**) | 0.375 |
|  | HA-100 | 1024 | 64 |  | ML-9 | 256 | 32 |  | Ripasudil | 1024 | 128 |  | Fasudil | 1024 | 128 |  | Hydroxyfasudil | 1024 | 128 |  |
| 17-R20 | TGC | 8 | 2(**4**) | 0.5 | TGC | 8 | 2 (**4**) | 0.5 | TGC | 8 | 2 (**4**) | 0.75 | TGC | 8 | 2(**4**) | 0.5 | TGC | 8 | 2 (**4**) | 0.75 |
|  | HA-100 | 1024 | 256 |  | ML-9 | 256 | 64 |  | Ripasudil | 1024 | 512 |  | Fasudil | 1024 | 256 |  | Hydroxyfasudil | 1024 | 512 |  |
| 14-R38 | TGC | 32 | 2 (**16**) | 0.5625 | TGC | 32 | 1 (**32**) | 0.53 | TGC | 32 | 2 (**16**) | 0.56 | TGC | 32 | 2 (**16**) | 0.56 | TGC | 32 | 2 (**16**) | 0.56 |
|  | HA-100 | 1024 | 512 |  | ML-9 | 512 | 256 |  | Ripasudil | 1024 | 512 |  | Fasudil | 1024 | 512 |  | Hydroxyfasudil | 1024 | 512 |  |
| 17-R108 | TGC | 8 | 2 (**4**) | 0. 5 | TGC | 8 | 2 (**4**) | 0.5 | TGC | 8 | 2 (**4**) | 0.75 | TGC | 8 | 2 (**4**) | 0. 5 | TGC | 8 | 2 (**4**) | 0.5 |
|  | HA-100 | 1024 | 256 |  | ML-9 | 256 | 64 |  | Ripasudil | 1024 | 512 |  | Fasudil | 1024 | 256 |  | Hydroxyfasudil | 1024 | 256 |  |
| 17-R39 | TGC | 16 | 2 (**8**) | 0.625 | TGC | 16 | 2 (**8**) | 1.125 | TGC | 16 | 2 (**8**) | 0.625 | TGC | 16 | 2 (**8**) | 0.625 | TGC | 16 | 2(**8**) | 0.625 |
|  | HA-100 | 1024 | 512 |  | ML-9 | 256 | 256 |  | Ripasudil | 1024 | 512 |  | Fasudil | 1024 | 512 |  | Hydroxyfasudil | 1024 | 512 |  |

TGC: tigecycline; Ripasudil: Ripasudil hydrochloride dihydrate; Fasudil: Fasudil hydrochloride; Hydroxyfasudil: Hydroxyfasudil hydrochloride. COMB: combination; FICI: fractional inhibitory concentration index. Synergy was defined as FICI ≤ 0.5, indifference as 0.5＜FICI ≤ 4, and antagonism as FICI > 4.
